# A novel Si/Sn composite with entangled ribbon structure as anode materials for lithium ion battery

**DOI:** 10.1038/srep29356

**Published:** 2016-07-08

**Authors:** Jinbo Wu, Zhengwang Zhu, Hongwei Zhang, Huameng Fu, Hong Li, Aimin Wang, Haifeng Zhang

**Affiliations:** 1 Shenyang National Laboratory for Materials Science, Institute of Metal Research, Chinese Academy of Sciences, 72 Wenhua Road, Shenyang, 110016, China

## Abstract

A novel Si/Sn composite anode material with unique ribbon structure was synthesized by Mechanical Milling (MM) and the structural transformation was studied in the present work. The microstructure characterization shows that Si/Sn composite with idealized entangled ribbon structured can be obtained by milling the mixture of the starting materials, Si and Sn for 20 h. According to the calculated results based on the XRD data, the as-milled 20 h sample has the smallest avergae crystalline size. It is supposed that the flexible ribbon structure allows for accommodation of intrinsic damage, which significantly improves the fracture toughness of the composite. The charge and discharge tests of the as-milled 20 h sample have been performed with reference to Li^+^/Li at a current density of 400 mA g^−1^ in the voltage from 1.5 to 0.03 V (*vs* Li/Li^+^) and the result shows that the initial capacity is ∼1400 mA h g^−1^, with a retention of ∼1100 mA h g^−1^ reversible capacity after 50 cycles, which is possible serving as the promising anode material for the lithium ion battery application.

Lithium ion batteries have been successfully applied as one of the most efficient power sources for many portable electronic devices due to their tremendous advantages, such as fast charging/discharging rate, high power performance, high stability, long cycle life, environment friendly and safety^1^,^2^. Recently, researches into large-scale lithium ion batteries have been ramped up for application of electric automobile, also as large-scale backup power supplies for grid energy storage^3^. The conventional commercialized lithium ion batteries use carbonaceous materials as the anode materials^4^,^5^ (e.g. carbon and natural graphite *et al.*). However, the theoretical specific capacity is limited at 372 mA h g^−1^ for LiC_6_ because of the intercalation mechanism, thus it cannot be able to meet the requirements of high power density and high specific capacity for plug-in hybrid and electric vehicles^6^,^7^,^8^.

Numerous investigations about possible anode materials have been undertaken to develop novel anode materials in order to replace the carbonaceous materials and fulfil the ever increasing demand of high performance lithium ion batteries^9^,^10^,^11^,^12^,^13^,^14^,^15^,^16^,^17^,^18^. Among the candidates, Si is considered to be the most promising anode material to take the place of the carbonaceous materials because of its high theoretical gravimetric capacity, about 4200 mA h g^−1^ (Li_22_Si_5_), an order of magnitude higher than that of conventional carbonaceous anode^19^,^20^,^21^. Unfortunately, intrinsic Si as an anode material for lithium ion battery has faced technical problem; the practical application of Si anode material is hindered by the large structural and volumetric change during charging and discharging process, being about 300–400%, which results in opening cracks and pulverization of the electrode and subsequent loss of electrical contact between the active material and current collector, leading to the final result of poor reversibility and rapid capacity fading^10^,^22^,^23^.

Significant efforts have been devoted to alleviate the volumetric expansion and contraction of Si during the lithiation and delithiation, including fabricating Si/C composite^24^,^25^, developing different Si alloy electrodes that contain another inactive buffer phase^26^. Another approach to counter the volume expansion problem is to develop nano-scaled materials. A number of various dimensional nano-scaled morphologies have been explored with the aim to minimize electrode pulverization and capacity loss in Si anodes using the nano size effect, such as nano-particles, nano-wires, nano-tubes and thin films^27^,^28^,^29^, which leads to limited improvement. However, the intrinsic mechanical properties of Si based anode materials have been neglected unintentionally so far. Indeed, from a micro-scopical point of view, the internal stresses anode suffered from the expansion and contraction during charge and discharge seems to have submitted to tensile and compressive loadings, the catastrophic failure of the Si anode is mainly due to lack of substantial toughness and damage tolerance. Therefore, the most fundamental issue is the fracture brittleness of the rigid Si which results in the cracks propagation. In fracture mechanics terms, the process of fracture can be considered as intrinsic damage processes and extrinsic mechanisms, hence the fracture toughness can be improved by intrinsic toughening and extrinsic toughening.

Extrinsic mechanisms can be quite diverse, involving such processes as cracking bridging by unbroken fibres or a ductile phase in the composites^30^.

In the present work, we focus on the mechanical characteristics of the anode composite and propose to consider the extrinsic toughening and use the lithium active material Sn (992 mA h g^−1^ for Li_22_Sn_5_), which is rather ductile hence able to withstand high deformation. A novel Si/Sn composite with entangled ribbon structure was synthesized by low-cost mechanical milling (MM)^31^,^32^,^33^.The detailed observation of the microstructure of the as-prepared composite was carried out. It can be seen Si particles were entangled within the Sn micro-ribbons. The additive Sn entangled ribbons can constrain the expansion of Si and tolerate the structural damage, which leads to the facile strain relaxation in the composite. It ensures the well electrical contact and mechanical integrity, leading to improvement of cycling stability as well as high reversible capacity. The electrode made by 80% nano-ribbon composite with 10% carbon black and 10% PVDF showed a high initial specific capacity about 1400 mA h g^−1^ with maintaining a steady reversible capacity about 1100 mA h g^−1^ after 50 cycles. The improved performance of the electrode with the entangled ribbon Si/Sn anode composite is probably due to the buffer effect of the ribbon type micro-structure. This anode is supposed to be one of the promising anode candidates for lithium ion batteries.

## Results

**Figure 1:**
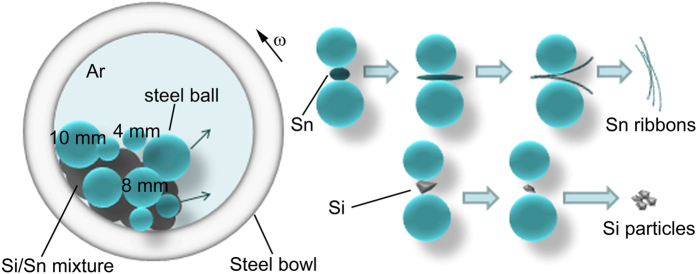
Schematic illustration of the formation processes of the Si/Sn composite with entangled ribbon structure during the mechanical milling.

Brittle Si and ductile Sn were chosen as the starting materials for preparation of the Si/Sn composite. Detailed processes were described in the Methods section. The mechanical milling was chosen to synthesize the Si/Sn composite because of its simplicity to enable the formation of the Sn entangled ribbon and the refinement of the rigid Si particles. Figure 1 is the schematic illustration of the formation processes of the entangled ribbon structure Si/Sn composite during the mechanical milling. As the steel bowl rotates at a speed of 300 rpm, the steel balls drop onto the powder arbitrarily. The ductile Sn and rigid Si powders can be trapped between the interfaces when the steel balls hit the chamber wall or two steel balls collide into each other. The raw material ductile Sn powders are flattened, fractured and welded repeatedly by ball milling. At the initial stage of formation, the high-energy impacts deform and unfold the ductile Sn. These deformed Sn powders weld onto the steel balls and the chamber walls at the second stage. During the repeated fractured and welded processes enforced by the ball milling, the deformed Sn powders can be ripped into different avulsions along with the cracks. Consequently, the deformation of Sn powders cause continuous size reduction and lead to formation of entangled ribbons. The unique entangled ribbon structure of the as-milled composite is probably due to the flexibility and the ductility of Sn and high impact energy of mechanical milling. As for the formation of the novel structure Si faceted particles, the repeated fractured and refinement processes enforced by the ball milling are different to the ductile Sn. It is well known that Si is the brittle non-metallic material which can be easily fractured and crushed. When the steel balls hit against the rigid Si particles, high energy impact results in direct pulverization of Si particles.

**Figure 2:**
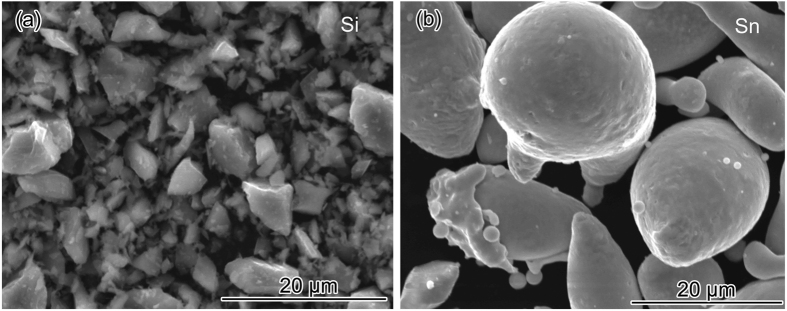
(**a**) SEM image of the precursor Si powders; (**b**) SEM image of the pristine Sn powders.

Figure 2a shows the SEM image of the precursor Si powders and Fig. 2b displays the SEM image of the pristine Sn powders. From the general particle views, the edges and corners of the brittle non-metallic Si particles can be clearly detected and the average particle size is less than 10 μm. In a sharp contrast to raw Si powders, the original ductile metallic Sn particles have spherical type or spheroid type morphological characteristic with a larger average particle size than raw Si powders, around 20 μm.

Five samples with specific milling time (1 h, 10 h, 15 h, 20 h, 25 h,) were prepared to study the structural transformation of the Sn entangled ribbon Si based composite during the milling processes. The phase transformation and characteristic of all the five as-milled composites were investigated by means of SEM.

**Figure 3:**
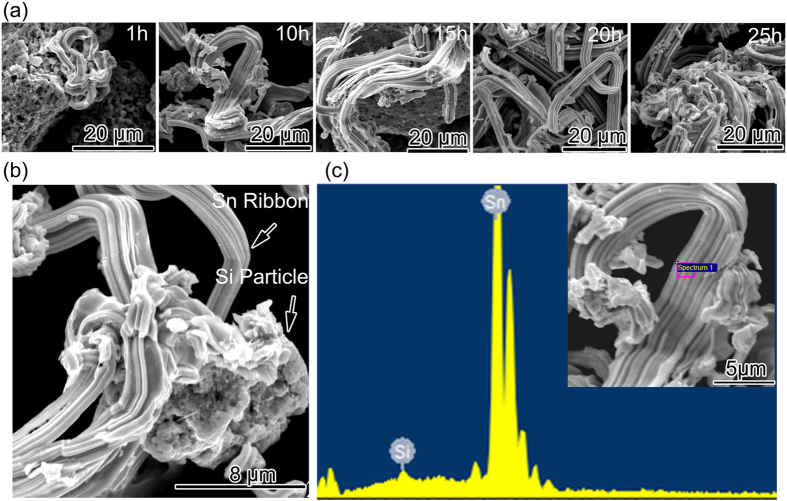
(**a**) SEM images of the as-milled 1 h, 10 h, 15 h, 20 h, 25 h samples; (**b**) High- magnification SEM image of the as-milled 20 h composite; (**c**) EDS pattern of the as-milled 20 h composite, inset picture is the SEM micrograph of the morphology.

Figure 3a displays the morphological transformation of the as-milled samples. The SEM images clearly reveal that the entangled ribbon structure appears at the first hour of ball milling and the number of the entangled ribbons keeps increasing with the time development till the milling time reaches to 20 h. After peaking at 20^th^ h, the number of the ribbons decreases because of the occurrence of large ribbons aggregation. The as-milled 20 h sample has the most entangled ribbons compared with the other four samples with different milling time and over milling results in growth of the ribbon cluster especially after 20 h, which indicates that 20 h may be the balance value for structural change. At the stage of 20 h, the formation of ribbons is equal to the decrease. With the aim to figure out the morphological features of the entangled ribbon, the as-milled 20 h sample was chosen to study the detailed morphology since it has the most entangled ribbons. More detailed microstructure information of the as-milled 20 h sample was given out in Fig. 3b. The SEM image clearly shows that faceted particle attaches to the ribbon and it is wrapped by the ribbon. Actually, the faceted particle is a cluster made of many smaller particles. It is assumed that the entangled ribbon is formed by metallic ductile Sn, and the faceted particle is the cluster made of rigid Si particles. The EDS analysis was carried out to confirm the hypothesis and identify the composition of entangled ribbons. Figure 3c presents the EDS image of the as-milled 20 h composite. From the inset image in Fig. 3c we can see that the ribbons have a thickness about 100 nm and a length up to several micrometers and the width of the ribbon is about 5 μm. The EDS pattern in Fig. 3c reveals that the entangled ribbon consists largely of ductile Sn.

**Figure 4:**
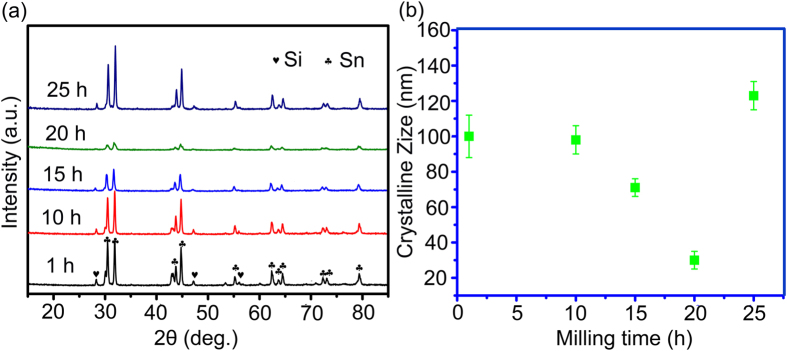
(**a**) XRD patterns of the five as-milled samples; (**b**) The calculated average crystalline size of the as-prepared five samples.

The X-ray diffraction patterns of the five as-milled samples are presented in Fig. 4a. It can be clear seen that the XRD pattern of every as-prepared composite is purely composed of the diffraction peaks of the element state Si and Sn. All the five as-milled composites demonstrate that there is no diffraction peaks corresponding to Si carbide and steel compounds can be observed on the patterns. During the milling process, the milling stopped and had 30 minutes break every one hour, which can minimize the possible thermal effect and prevent the oxidation of Si and Sn. Hence, the HEMM would not induce Si oxide compounds and other metallic contamination. In addition, it is interesting to find out from 1^th^ h to 20^th^ h, the diffraction peak intensity goes weaker with the milling time growth. The peak intensity decreases to the lowest point when the total milling time increases to 20 h. After that, the diffraction peaks go stronger accompanied by the time growing. Generally, the structural and morphological transformation can be the reason for the intensity change of the diffraction peaks. It means that there must be some structural transformation during the milling process, including the crystalline size and morphology. From the information given by the XRD patterns, especially the full width at half maximum (FWHM) of the diffraction peaks, the average grain size was calculated according to the Scherrer formula: crystallite size = kλ/β cos θ, using Traces Program. The calculated average crystalline size of the as-prepared five samples is shown in Fig. 4b. As we can see the evolution of the grain size is in good agreement with the development of the XRD patterns. The crystalline size is maintained at around 100 nm in the period from the beginning of milling to 10^th^ h. However, after 10^th^ h, the crystalline size decreases dramatically until the milling time reaches to 20^th^ h. At the time of 20^th^ h, the composite has a minimum crystallite size of about 30 nm. However, over-milling results in the growth of the crystalline size. The crystalline size of the Si/Sn composite grows to about 120 nm, larger than the initial stage.

**Figure 5:**
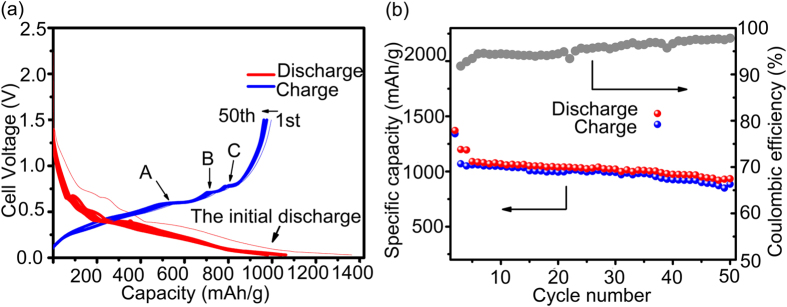
(**a**) Charge–discharge curves of the as-milled 20 h entangled ribbon Si/Sn composite electrode between 0.03 V and 1.5 V at a current of 500 mA g^−1^; (**b**) Cycling performance of the as-milled 20 h Si/Sn composite electrode. The electrode was cycled at at 0.1 C in the voltage from 1.5 to 0.03 V for 50 cycles.

Figure 5a shows the discharging (lithiation) and charging (deliathiation) profiles of the as-milled 20 h Sn entangled ribbon Si based composite electrode under a controlled level at 500 mA g^−1^ analyzed for up to 50 cycles. The as-measured sample delivers high initial specific capacity approaching about 1000–1370 mA h g^−1^ (charging-discharging) with an initial irreversible capacity values around 370 mA h g^−1^, which means that the first cycle coulombic efficiency has a value of 77%. The initial irreversible capacity is believed to be attributed by the formation of the solid electrolyte interface (SEI). Also, some Li^+^ was trapped by oxide and formed Li oxide (Li_2_O) which consumed Li^+^ and cause initial capacity loss. There are some potential plateaus (See arrow A, B and C) on the charging curves at the voltage between 0.5 V and 1 V, which corresponds to the delithiation of ductile Sn. Si has lower sloping lithiation/delithiation potential plateau from 0.4 until 0.1 V compared with Sn, hence they can act as the buffer frame matrix for each other during Li^+^ incorporation/removal, which can accommodate volume change and tolerate large stress evolution^34^. The discharging (lithiation) and charging (deliathiation) profiles show high coincidence from 2^nd^ cycle to 50^th^ cycle with the discharging capacity maintaining at over 1000 mA h g^−1^ indicating a good Li insertion and extraction reversibility. More detailed study of the electrochemical properties and performance of the Sn entangled ribbon Si based composite are carried out by cycling test. Figure 5b illustrates the cycling capacities and coulombic efficiency as a function of cycle number at 0.1 C in the voltage from 1.5 to 0.03 V. The Sn entangled ribbon Si based based composite exhibits a high initial discharge capacity of 1400 mA h g^−1^, which decreases to 1200 mA h g^−1^ at the second cycle, with a first cycle efficiency of 85.7%. It is worthy of notice to see that the coulombic efficiency quickly recovers to around 100% after the first five cycles. The capacity retention is quite stable, maintaining around 1100 mA h g^−1^ for the subsequent cycles.

## Discussion

The novel entangled Sn ribbon Si based composite is used as the anode material for lithium ion battery. It is supposed that this entangled ribbon structure can help enhance the damage tolerance. As the active Si particles are wrapped by the Sn entangled ribbons, substantial toughness of the whole combination is optimized compared with the purely intrinsic Si particle. The integrity of the electrical contact between active Si particle and the Cu current collector can be maintained even if the fracture of the brittle Si particles happens during the lithiation and delithiation.

**Figure 6:**
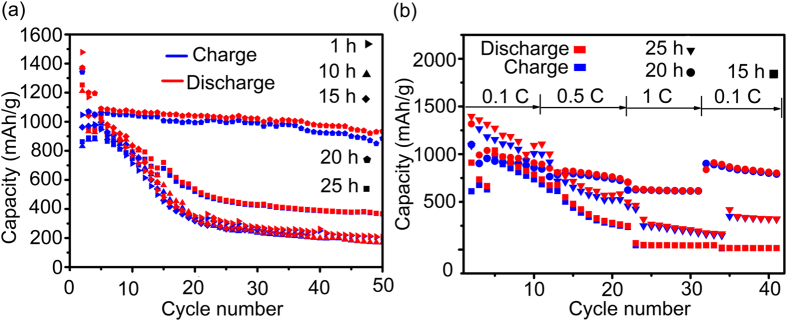
(**a**) Comparison of cycling performance of the five as-milled Si/Sn composite electrodes, all the electrodes were cycled at 600 mA g^−1^ for 50 cycles; (**b**) The corresponding rate capability of the as-milled 15 h, 20 h and 25 h Si/Sn composite electrodes cycled at various rates of 0.1, 0.5 and 1 C for total 40 cycles.

Figure 6a confirms the unique contribution made by the entangled ribbon structure. The cycle properties of the five as-milled Si based anodes (1 h, 10 h, 15 h, 20 h, 25 h) are compared under the controlled level of 600 mA g^−1^. The as-milled 1 h sample exhibits the highest initial discharge capacity of 1500 mA h g^−1^ but dramatically fades to 500 mA h g^−1^ after 15 cycles and then continuously drops to 200 mA h g^−1^ after 50 cycles. The as-milled 10 h and 15 h samples show similar specific capacity variation tendency but with lower capacity than the as-milled 1 h sample. The as-milled 10 h sample delivers an initial capacity of 1200 mA h g^−1^ while the as-milled 15 h sample displays an initial capacity of about 1400 mA h g^−1^. Unfortunately, both of them drop to 200 mA h g^−1^ after 50 cycles. As for the as-milled 20 h sample which has the most entangled ribbons, an initial discharge capacity of 1370 mA h g^−1^ presented, and then the discharge capacity decreases to 1100 mA h g^−1^ at the 2^nd^ cycle. However the as-mill 20 h Si/Sn composite shows stable cycling performance with the capacity maintaining at 1000 mA h g^−1^ in the subsequent 50 cycles. Such a result indicates that the electrochemical performance of the as-milled 20 h Si/Sn composite which has the most entangled ribbons is improved compared with the other four as-milled samples with less ribbons. The stable cycling performance of the as-milled 20 h Si/Sn composite anode electrode is supposed to be beneficial from the entangled ribbon structure of ductile Sn which can serve as an immobile and conductive framework to maintain the electrical contract between the active Si and the Cu current collector, hence prevents the polarization of the electrode during the Li^+^ incorporation and removal. For the anode electrode prepared from the over milling sample (as-milled 25 h), it delivers 1250 mA h g^−1^ at the first cycle and then linearly declines to 700 mA h g^−1^ after 15 cycles, finally fades to 400 mA h g^−1^ after 50 cycles. This over-milling sample has better cycling performance than the as-mill 1 h, 10 h and 15 h samples but worse than the as-milled 20 h Sn entangled ribbon Si based composite.

Further investigation has been done to analyze the influence of the entangled ribbon structure to the rate capability of the as-milled Si/Sn composite. The electrodes prepared from as-milled 15 h, 20 h and 25 h samples charged and discharged at predetermined C rate (0.1 C, 0.5 C and 1 C) in the voltage arrange from 1.5 to 0.03 V. Figure 6b shows the variation of the discharge specific capacities versus cycle numbers. As shown, the electrode prepared using as-milled 20 h Si/Sn composite operates with a more stable response than the electrodes prepared using as-milled 15 h and 25 h samples relative to C rate change. For instance, the electrode using as-milled 20 h Sn entangled ribbon Si based composite exhibits the average discharge specific capacity of about 1000 mA h g^−1^ at 0.1 C in the first 10 cycles, and it declines slightly to 900 mA h g^−1^ when the C rate increases to 0.5 C in the following 10 cycles. Even at 1 C in the subsequent 10 cycles, the electrode can still maintain a stable average capacity about 700 mA h g^−1^. In contrast, for the case of the electrode using as-milled 15 h sample and over-milling sample (as-milled 25 h), the capacity fade rapidly when the C rate increases to 0.5 C in the second 10 cycles. Even worse, they can only deliver less than 250 mA h g^−1^ at 1 C in the third 10 cycles. Once upon changing the C rate from 1 C to 0.1 C in the last stage, the specific capacity of the electrode using as-milled 20 h Si/Sn composite which has the most entangled ribbons could be restored to be closed to its original value about 1000 mA h g^−1^, but the capacity of the other two electrode could only be restored to less than 500 mA h g^−1^.

The as-milled 20 h Sn entangled ribbon Si based composite shows the excellent electrochemical performance should be attributed to the following reasons: firstly, during the charge and discharge, the entangled ribbon structure have more space can accommodate the expansion of active Si than the pure bulk Si, which alleviates the intrinsic stress. Secondly, Si and Sn has different potential plateau, which provide a buffer to the volume effect for each other. Thirdly, the entangled ribbons can also act as the constrained frame for the pulverization of Si and maintain the integrity of the electrical contact between the active material and the current collector.

## Methods

### Materials synthesis

Planetary ball milling (Fritsch P5) was used to fabricate the Si/Sn composite. The commercial Si powder (≥99.99%, ∼300 mesh) and Sn powder (≥99.5%, ∼200 mesh) were used as starting materials. 50 at% Si powder and 50 at% Sn powder were mixed according to the predetermined nominal composition Si_50_Sn_50_ and sealed into a 250 mL tempered steel bowl with bearing steel balls. Bearing steel balls with different diameters were mixed in one bowl during the milling. With this configuration, the space between the balls can be reduced, which can make sure of the completely and uniformly milling. The ball to powder mass ratio was 10:1. Mechanical milling was carried out in a Fritsch P5 planetary mill under argon atmosphere with 30 minutes break every hour in order to remove the possible thermal effect. The mechanical milling has a high milling frequency with a rotation speed of about 300 rpm. Samples with different ball milling time were prepared to observe the evolution of the material structure.

### Structural Characterization

X-ray diffraction (XRD) patterns of the as-synthesized composites were obtained using a Rigaku D/max-2500pc diffractometer (reflection 2 theta geometry, Cu *kα* radiation). The morphologies of the starting material and the microstructure characteristic and element analysis of the as-prepared composites were examined by Quanta 600 scanning electron microscope (SEM) equipped with Oxford energy dispersive X-ray spectrometry (EDS).

### Electrochemical measurements

Electrochemical performance was evaluated using CR2032-type coin cells. The composite materials working electrodes were prepared by coating the slurries onto the Cu foil (a thickness of ∼25 μm). The slurry was fabricated by mixing 80 wt% active composite materials, 10 wt% carbon black (Super-p) and 10 wt% poly-vinylidene fluoride (PVDF). After coating, the film was dried at 120 °C for 24 h under vacuum to remove the water, then cut into sheets with 12 mm in diameter and compressed under a pressure of 2 × 10^5^ Pa between two stainless steel plates. Lithium metal was used as the Li^+^ source, counter and reference electrode. 1 M LiPF_6_ (dissolved in ethylene carbonate and dimethyl carbonate with a 1:1 volume ratio) was applied as the electrolyte in this work. A sheet with 16 mm in diameter Celgard 2400 membrane was utilized as the separator. The coin cells were assembled in an argon-filled glove box with the content of oxygen and moisture below 1 ppm. For comparison, samples with different milling time were used as the active materials to prepare the electrodes in a similar way. All the electrochemical tests (including C rate and cycling test) were performed using a LAND-CT2001A battery test system (Jinnuo Wuhan Corp., China) in the voltage from 0.03 to 1.5 V versus (Li^+^/Li) at room temperature. Different current densities were used during the C rate test.

## Additional Information

**How to cite this article**: Wu, J. *et al.* A novel Si/Sn composite with entangled ribbon structure as anode materials for lithium ion battery. *Sci. Rep.* **6**, 29356; doi: 10.1038/srep29356 (2016).
